# Potential short-term outcome of an uncontrolled COVID-19 epidemic in Lombardy, Italy, February to March 2020

**DOI:** 10.2807/1560-7917.ES.2020.25.12.2000293

**Published:** 2020-03-26

**Authors:** Giorgio Guzzetta, Piero Poletti, Marco Ajelli, Filippo Trentini, Valentina Marziano, Danilo Cereda, Marcello Tirani, Giulio Diurno, Annalisa Bodina, Antonio Barone, Lucia Crottogini, Maria Gramegna, Alessia Melegaro, Stefano Merler

**Affiliations:** 1 Bruno Kessler Foundation, Trento, Italy; 2 Lombardy Region, Directorate General for Health, UO Prevenzione, Milan, Italy; 3 Health Protection Agency of Pavia, Department of Hygiene and Preventive Medicine, Pavia, Italy; 4 Bocconi University, Dondena Centre for Research on Social Dynamics and Public Policy, Milan, Italy; 5 These authors are joint senior authors and contributed equally to this work.

**Keywords:** COVID-19, SARS-CoV-2, Coronavirus, Lombardy outbreak, modelling

## Abstract

Sustained coronavirus disease (COVID-19) transmission is ongoing in Italy, with 7,375 reported cases and 366 deaths by 8 March 2020. We provide a model-based evaluation of patient records from Lombardy, predicting the impact of an uncontrolled epidemic on the healthcare system. It has the potential to cause more than 250,039 (95% credible interval (CrI): 147,717–459,890) cases within 3 weeks, including 37,194 (95% CrI: 22,250–67,632) patients requiring intensive care. Aggressive containment strategies are required.

On 20 February 2020, a case of coronavirus disease (COVID-19) was notified in Lombardy, Italy, uncovering ongoing transmission in at least two other regions (Emilia Romagna and Veneto) [1]. To rapidly assess risks of the current situation, we analysed the line list of reported cases in Lombardy to project the number of cases, should the epidemic be left uncontrolled.

## Epidemic projections

We projected the number of COVID-19 cases in 593 municipalities of Lombardy where at least one case of community transmission (i.e. excluding cases in healthcare workers or known nosocomial exposure) had been recorded by 8 March 2020. These represented 39.4% of all municipalities in the region and a total population of ca 6.9 million inhabitants (68.8% of the Lombardy population). The projections were based on a stochastic susceptible-infectious-removed (SIR) transmission dynamic model for each municipality and assuming that no control measures were in place. The model considered a population structured in 20 age groups (19 5-year age groups from 0 to 94 years and one age group for ≥ 95-year-olds) according to municipality-specific age distributions [2]. The Polymod contact matrix for Italy was incorporated to simulate the heterogeneity of contacts by age [3]. The model considered three consecutive infectious compartments to simulate a gamma distributed generation time of a mean of 6.6 days [1], longer than estimates obtained for Chinese provinces outside Hubei [4]. R_0_ was sampled from the posterior distribution estimated for Lombardy: mean: 3.1, 95% credible interval (CrI): 2.9–3.2 [1]. We assumed asymptomatic and symptomatic individuals to be equally infectious as shown by preliminary analysis of virological data from the same region [1].

We considered age-specific reporting and severity rates, which were estimated from the Lombardy line list. In particular, the fraction of reported infections at different ages *r(a)* was defined as

$$r(a)=r_{m}\frac{p(a)}{i(a)}$$

where *r_m_* represents the overall fraction of reported infections in the population; *i(a)* is the proportion of expected infections in age *a* and *p(a)* is the proportion of cases reported in age group *a*. We computed *i(a)* as the average age distribution of cases at the end of 500 simulated epidemics in the model.

Note that *r(a)* represents the probability that an infection occurring in age *a* is reported, while *p(a)* represents the contribution of reported cases of age *a* to the overall amount of reported cases. We estimated *p(a)* for seven age groups (0–19, 20–29, 30–39, 40–49, 50–59, 50–69 and ≥ 70 years). Only seven age groups were considered here in order to analyse a sufficiently large sample of reported cases for each age group. Estimates of *p(a)* were obtained by a Markov chain Monte Carlo (MCMC) sampling, applied to the multinomial likelihood of observing the reported number of cases *C(a)* in age group *(a)*:

$$L=\frac{C!}{\prod_{a}C(a)!}\prod_{a}p{\left(a\right)}^{C(a)}$$

where *C* is the overall number of positive individuals. Specifically, the posterior distribution of *p(a)* was computed by using the random-walk Metropolis–Hastings algorithm based on normal jump distribution, which represents a common method to generate sequence of samples from an unknown probability distribution [5].

The relative risk of developing critical disease by age, *z(a)*, was assumed to be proportional to the fraction of critical and deceased cases observed in the corresponding age group, *v(a)*: :

$$z\left(a\right)=\frac{\nu \left(a\right)}{\sum_{a}p\left(a\right)\nu \left(a\right)}$$

The proportion of reported cases of age *a* who develop critical disease, *w(a)*, is given by *w(a) = w_m_z(a)*, where *w_m_* represents the overall fraction of critical cases among reported cases. In the projections, we assumed *r_m_* = 9.2% (95% CrI: 5–20%) for the overall reporting rate [6] and *w_m_* = 15% for the overall proportion of reported cases requiring intensive care [1].

**Figure 1:**
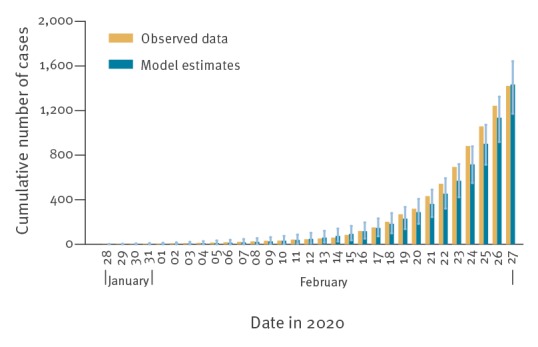
Observed and estimated cumulative number of reported coronavirus disease cases aggregated over 593 municipalities (population 6.9 million), Lombardy, 1–27 February 2020 (n = 1,400 observed cases)

For each municipality, we initialised the model with a fully susceptible population and a single infectious individual, of age chosen with probability proportional to its age structure. Since we do not know when the initial infection was introduced in each municipality, we start our model with one infectious individual at day 1 of the simulation time. At a given day *t* of the simulation time, the cumulative number of confirmed cases in the model reaches the cumulative number of confirmed cases observed on 27 February in that municipality. We then assumed that day *t* of the simulation time corresponds to the calendar day 27 February, dated back the day of first introduction accordingly and projected the number of cases between 9 and 28 March 2020. Data reported in the line list after 27 February may still be incomplete because of reporting delays [1]. We filtered out all simulations that ended in stochastic extinction of the epidemic before 27 February and we ran the model until we had 500 valid simulations for each municipality. A validation of the model is shown in Figure 1.

Vertical lines represent 95% credible intervals of model estimates.

**Figure 2:**
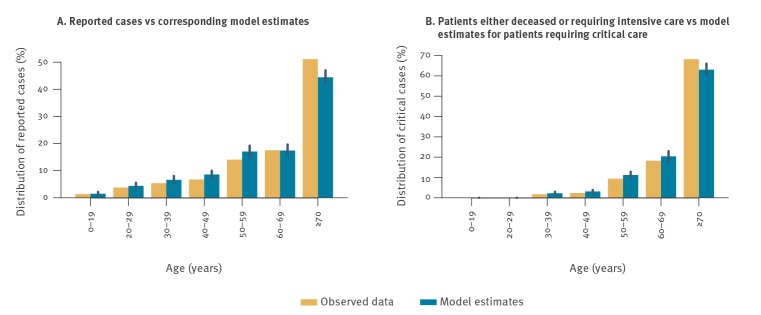
Age distribution of reported cases (n = 1,400) and of patients either deceased or requiring intensive care (n = 278), compared with corresponding model estimates, Lombardy line list, 1–27 February 2020

**Figure 3:**
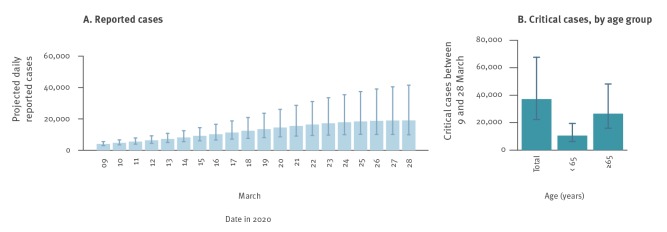
Projected daily number of reported and critical cases, aggregated over 593 municipalities, Lombardy, 9 and 28 March 2020

The observed and modelled age distribution of reported and critical cases is shown in Figure 2. About 63% (95% CrI: 60–66%) of critical cases were estimated to be individuals older than 70 years. In the absence of any intervention, we estimated that the number of new cases per day during the period 9–28 March would reach 19,060 (95% CrI: 9,898–41,491). A total of 250,039 (95% CrI: 147,717–459,890) reported cases were expected to occur between 9 and 28 March in the considered population of Lombardy (Figure 3). During the same period, the model estimated a total of 37,194 (95% CrI: 22,250–67,632) patients requiring critical care. As a comparison, the total number of COVID-19 cases recorded in the Lombardy line list by 23 March 2020 (in the presence of interventions) was 28,761, 20,043 of whom were symptomatic cases. 

Vertical lines represent 95% credible intervals of model estimates.

Bars: mean estimates; vertical lines: 95% of credible interval associated with uncertainties in values of R_0_, the overall reporting rate* r_m_*, as well as the variability of the stochastic model realisations.

## Discussion

Following the notification of a COVID-19 case in Lombardy on 20 February 2020, which triggered testing and detection of autochthonous transmission in at least two other regions (Emilia Romagna and Veneto), immediate control measures were adopted by regional and national governments, including the institution of quarantined zones on 23 February enclosing more than 50,000 inhabitants. Despite these measures, the spatial distribution of the epidemic expanded rapidly throughout the country. By 8 March, all Italian regions had reported at least one case, with seven regions reporting more than 100 cases and nine regions reporting at least one death. In Lombardy, the regional health authority started to fill in a line list of COVID-19-positive cases [1], comprising 5,830 infections, with 5,626 confirmed symptomatic cases and 588 infections recorded among healthcare workers as at 8 March.

The ongoing COVID-19 outbreak in Italy and, particularly, in the Lombardy region has put the healthcare system under massive strain, given the rapid epidemic spread and the need to provide intensive care support to an increasing number of patients. Healthcare systems and policymakers had to face rapid decisions to contain the contagion process and limit the number of fatalities. Here we presented epidemic projections assuming no interventions and R_0_ = 3.1. This value of the basic reproduction number was estimated in Cereda et al. [1], analysing the exponential phase of the epidemic in Lombardy; it compares well to the estimates obtained during the early epidemic phase in Wuhan, China [7].

The still limited knowledge on COVID-19 epidemiology in Italy certainly represents a limitation of this study as some parameters such as the overall reporting rate and the overall proportion of reported cases requiring intensive care were estimated in the Chinese context [4,6]. Based on the analysis of Chinese contact data, we considered that children are as likely to be infected as adults [8,9]. Moreover, we assumed no differences in infectiousness between symptomatic and asymptomatic infected individuals. Although this choice was supported by the analysis of virological data presented by Cereda et al. [1], further evidence is required to fully validate this hypothesis. Finally, we did not consider potential temporal trends in the reporting rate and delays in the development of symptoms requiring intensive care. It is also possible that our projections underestimate the potential impact of an uncontrolled epidemic, as we do not allow for contacts between individuals in different municipalities (which may re-ignite transmission in locations where the infection becomes stochastically extinct) and we assumed that the infection did not spread further to other municipalities inside and outside Lombardy.

Even in the presence of the above limitations, the simulated cumulative number of cases between 28 January and 27 February well compares with data (for each considered day, data points fall in the credible interval of model estimates). The presented results show that in a scenario of uncontrolled transmission, the number of severe and critical cases will become largely unsustainable for the healthcare system in a very short time. Drastic governmental interventions and widespread behavioural changes of the population are required to limit COVID-19 transmission and avoid catastrophic effects on the healthcare system.
